# The therapeutic value of *Myrtus* communis *L.*: an updated review

**DOI:** 10.1007/s00210-024-02958-3

**Published:** 2024-02-06

**Authors:** Ali Esmail Al-Snafi, John Oluwafemi Teibo, Hazem M. Shaheen, Opeyemi Abigail Akinfe, Titilade Kehinde Ayandeyi Teibo, Numonde Emieseimokumo, Mohamed M. Elfiky, Hayder M. Al-kuraishy, Ali I. Al-Garbeeb, Athanasios Alexiou, Marios Papadakis, Hitham Alaa Mohammed Mahana, Ahmed Maher Younes, Osama Ashraf Elbanna, Abd-elrahman Ali Radwan Qasem, Ibrahim Yasser Ibrahim Shahin, Gaber El-Saber Batiha

**Affiliations:** 1 Department of Pharmacology, College of Medicine, University of Thi-Qar, Nasiriyah, Iraq; 2 Department of Biochemistry and Immunology, Ribeirão Preto Medical School, University of São Paulo, Ribeirão Preto, São Paulo Brazil; 3 Department of Pharmacology and Therapeutics, Faculty of Veterinary Medicine, Damanhour University, Damanhour, 22511 AlBeheira Egypt; 4 Department of Biochemistry, University of Ibadan, Ibadan, Nigeria; 5 Department of Maternal-Infant and Public Health Nursing, College of Nursing, Ribeirão Preto, University of São Paulo, São Paulo, Brazil; 6 Department of Medical Biochemistry, Rivers State University, Rivers State, Port Harcourt, Nigeria; 7 Anatomy Department, General Medicine Practice Program, Batterjee Medical College, Jeddah, Saudi Arabia; 8 Anatomy Department, Faculty of Medicine, Menoufia University, Shibin El Kom, Egypt; 9 Department of Pharmacology, Toxicology and Medicine, Medical Faculty, College of Medicine, Al-Mustansiriyah University, P.O. Box 14132, Baghdad, Iraq; 10 University Centre for Research & Development, Chandigarh University, Chandigarh-Ludhiana Highway, Mohali, Punjab India; 11 Department of Research & Development, Funogen, Athens, 11741 Greece; 12 Department of Research & Development, AFNP Med, Wien, 1030 Austria; 13 Department of Science and Engineering, Novel Global Community Educational Foundation, Hebersham, NSW 2770 Australia; 14 Department of Surgery II, University Hospital Witten-Herdecke, University of Witten-Herdecke, Heusnerstrasse 40, 42283 Wuppertal, Germany

**Keywords:** *Myrtus communis*, Myrtle, Phytochemical, Pharmacology, Therapeutics

## Abstract

*Myrtus communis L.* (Family: Myrtaceae) is naturally found in the western part of Asia, Southern Europe, and North Africa. It has been reportedly applied in pharmaceutical industry, traditional medicine, cosmetics, spices, and food. Pubmed, Google scholar, Web of Science, and Scopus were utilized to seek out relevant content concerning the therapeutic potential of *M. communis*. Subsequently, we conducted a review to identity noteworthy updates pertaining to *M. communis*. Myrtle berries, leaves, seeds, and essential oils are natural sources of several nutrients and bioactive compounds with marked health effects. The chemical analysis showed that *M. communis* contained oils, alkaloids, flavonoids, phenolics, coumarins, saponosides, tannins, quinines, and anthraquinones. A pharmacological investigation revealed that *M. communis* possessed anti-inflammatory, analgesic, antimicrobial, antiparasitic, antioxidant, antidiabetic, anticancer, antimutagenic, immunomodulatory, dermatological, cardiovascular, central nervous system, and gastrointestinal protective effects, among numerous other biological effects. This current review focused on the biochemical, pharmacological, therapeutic effects, and various biological activities of different parts of *M. communis*. It signifies that *M. communis* is a therapeutic plant with numerous applications in medicine and could be used as a drug isolate based on its safety and effectiveness.

## Introduction

**Fig. 1:**
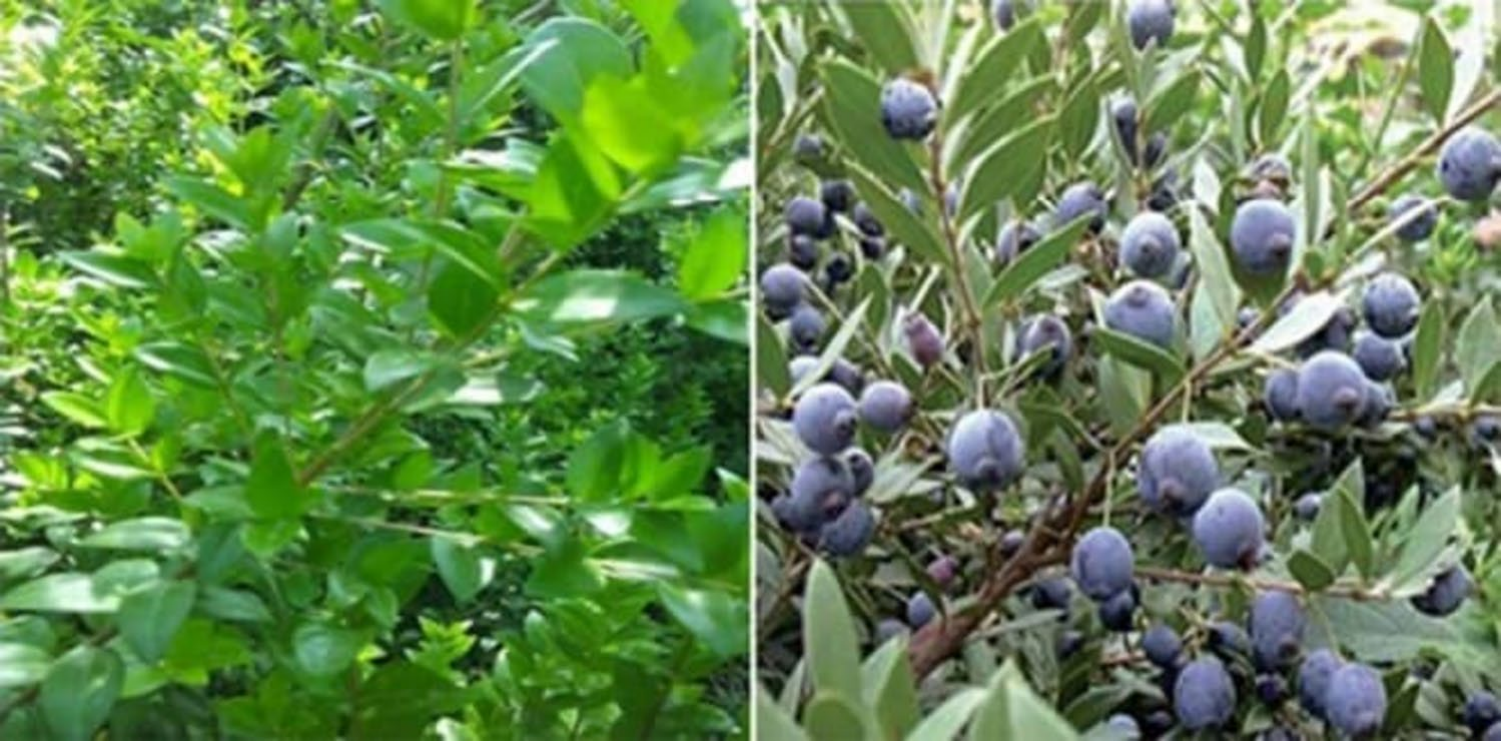
*M. communis plant and berries* (reproduced from Sisay and Gashaw 2017)

Myrtle (*Myrtus communis L.*) is an aromatic medicinal plant, typical of the coastal areas of the Mediterranean regions, such as North Africa or Southern Europe, but it is also present in South America, Australia, and in some areas of Himalaya (Alipour et al. 2014; Jabri et al. 2018; Sumbul et al. 2010, Gaber et al., 2021). It belongs to the Myrtaceae family, which includes about 3000 species, and grows spontaneously as an evergreen shrub or a small tree. The plant can reach a height of 2.5 m, with a full head deeply covered by branches and small leaves; flowers are starry, scented, and can be white or pink, whereas berry fruits are edible, small, with a round shape and many seeds inside, generally blue-black, even if some varieties have white-yellow fruits, and ripen in autumn, between October and February (Fig. 1).

Secondary metabolites are produced by biologically synthesizing the primary metabolites of plants which are the major composition of chemicals in agrochemicals, food additives, biopesticides, colors, fragrances, flavors, and pharmaceuticals (Al-Snafi 2013a, b; Al-Snafi 2014a, b; Al-Snafi 2016; Al-Snafi 2018; Salehi et al. 2019; Gaber et al; 2021; Al-Snafi 2021; Al-Snafi, Ibraheemi & Talab 2021).* M. communis* has been reportedly applied traditionally in medicine, spices, and food preparation. The chemical analysis showed that *M. communis* contained oils, alkaloids, flavonoids, phenolics, coumarins, saponosides, tannins, quinines, and anthraquinones. A pharmacological investigation revealed that *M. communis* possessed anti-inflammatory, analgesic, antimicrobial, antiparasitic, antioxidant, antidiabetic, anticancer, antimutagenic, immunomodulatory, dermatological, cardiovascular, central nervous system, and gastrointestinal protective effects, among numerous other biological effects.

Many plants that have therapeutic properties have recently been reviewed showcasing their biological and pharmacological properties and expanding their prospect in the drug development pipeline against the management of various diseases (Batiha et al., 2023a, b; Teibo et al. 2021).

A comprehensive search was carried out on Pubmed, Google scholar, Web of Science, and Scopus to seek out relevant content concerning the therapeutic potential of *M. communis*. Subsequently, we conducted a review to identity noteworthy updates pertaining to *M. communis* which was used for the study.

This current review focused on the biochemical and pharmacological constituents and therapeutic effects of *M. communis*.

## Taxonomic classification

**Table:** 

Kingdom	Plantae
Subkingdom	Viridiplantae
Infrakingdom	Stretophyta
Superdivision	Embryophyta
Division	Tracheophyta
Subdivision	Spermaophytina
Class	Magnoliopsida
Superorder	Rosanae
Order	Myrtales
Family	Myrtaceae
Genus	Myrtus
Species	Myrtus communis

### Distribution

*M. communis* has its origins in the western part of Asia, northern Africa, and southern Europe. It was dispersed across **Africa** (South Africa, Algeria, Ethiopia, Eritrea, Libya, Morocco, Tunisia), **Asia** (Yemen, Afghanistan, Cyprus, Iraq, Iran, Jordan, Syria, Lebanon, Turkey, Palestine, Pakistan), **Europe** (Albania, France, Greece, Portugal, Malta, Spain, Former Yugoslavia) (Aslam et al. 2010; Jabri et al. 2016a, b, c).

## Description

It is a bushy, strong-scented evergreen, upright shrub, about 3 m high, that arches as the year goes by. The leaves are in threes, simple, and have the shape ovate-lanceolate (Fig. 1). The leaf length is 2.5–5 cm, its color is dark green, and other features include aromatic when crushed; the glossy nature; short petioles; veins that are pinnated and glabrous; the flowers that are white or pink; solitary, axillary; and could be rose-tinged. It has the shape of a bowl, with a length of 2 cm. The plant is also actinomorphic, bisexual, and epigynous with slender pedicels of length 2 cm; the calyx is turbinate calyx tube of four to five sepals; the corolla is four to five petalled; fruit is oblong-ellipsoid white and 1 cm long (Bouzabata 2013). The berry fruits are edible, small, with a round shape and many seeds inside, generally blue-black, even if some varieties have white-yellow fruits, and ripen in autumn, between October and February. Insects do pollination, and birds spread seeds in the environment (Alipour et al. 2014; Petretto et al. 2016).

## Traditional uses

All the aerials’ parts, fruits, leaves, and essential oil are medicinal. The applications of myrtle are in food, spices, and traditional medicine. The decoction of myrtle aerial part was used as hypotensive, hypoglycemic, anti-inflammatory, and antidiarrhea in the treatment of bleeding, conjunctivitis, epistaxis, peptic ulcers, palpitation, urethritis, hemorrhoids, headache, leukorrhea, excessive perspiration, skin diseases such as pulmonary treating cough, gastrointestinal disorders (i.e., peptic ulcers, diarrhea, and hemorrhoids), urinary diseases (i.e., urethritis), and skin ailments (i.e., reddened skin), as well as for inactivating microorganisms and wound healing (Sumbul et al. 2010; Boudjelal et al. 2013; Aleksic and Knezevic 2013, Aleksic et al. 2014). Fruits have a wide range of applications: as astringent, analgesic, antiseptic, carminative, demulcent, anti-inflammatory, diuretic, antidiabetic antiemetic, nephroprotective, homeostatic, and for stomach disorders. Leaves were applied as flavoring agent, astringent, antiseptic, blood purifier hypoglycemic, laxative, analgesic, homeostatic, stimulant, and in the treatment of constipation and respiratory diseases (Sumbul et al. 2012; Dellaoui et al. 2018).

## Physicochemical characteristic

### *M. communis* berry

Myrtle berries are round fruits composed of a fleshy pericarp and a snail-shaped seed (Pezhmanmehr et al. 2010). Table 1 below shows the physicochemical properties of berries of *M. communis.*

**Table 1:** Physicochemical properties of berries of *M. communis*

Ash values	Value	Successive extractive values	Values	Cold extractives	Values	Hot extractive	Value
Acid insoluble ash	0.2 ± 0.02%	Chloroform	1.27 ± 0.04%	Chloroform	1.43 ± 0.05%	Methanol	25.10 ± 0.11%
Water soluble ash	2.4 ± 0.31%	Petroleum ether, 60–80 °C	1.03 ± 0.03%	Petroleum ether, 60–80 °C	0.64 ± 0.04%	Petroleum ether, 60–80 °C	1.14 ± 0.04%
Sulfated ash	3 ± 0.28%	Water	9.00 ± 0.23%	Methanol	11.2 ± 0.14%	Chloroform	2.05 ± 0.03%
Total ash content	5.6 ± 0.17%	Methanol	14.88 ± 0.10%	Water	19.17 ± 0.16%	Water	27.40 ± 0.06%

(Sumbul et al. 2012)

### *M. communis* seed

Since myrtle fruit is rich in secondary metabolites, the seeds in particular have previously been reported to contain a larger number of phenolic compounds in comparison with other tissues in the pericarp (Wannes and Marzouk 2013; Taamalli, et al. 2014b, c, a; Andrea et al. 2019). Ellagic acid is released after hydrolysis of ellagitannins, and its presence in myrtle berries is well documented (Taamalli et al. 2014b, c, a; Taamalli et al. 2014b, c, a). Two main dimeric ellagitannins, eugeniflorin D2 (Al-Snafi 2013a, b) and oenothein B (Al-Snafi 2013a, b), with a specific macrocyclic structure were isolated from seed extracts and chemically characterized (Table 2).

**Table 2:** Ellagic acid and ellagitannins in myrtle berry seeds

Samples (no. of specimens)	Ellagic acid Year 2008–2009	Eugeniflorin D_2_	Oenothein B
Cultivated	**0.48 ± 0.09**	**5.35 ± 2.10**	**13.61 ± 2.20**
Wild	**0.55 ± 0.15**	**5.14 ± 1.80**	**11.03 ± 1.13**
	**Year 2010–2011**		
Cultivated	**0.33 ± 0.09**	**4.91 ± 0.76**	**10.84 ± 2.50**
Wild	**0.40 ± 0.15**	**5.32 ± 1.23**	**10.55 ± 3.07**
	**Year 2013–2014**		
Cultivated	**0.65 ± 0.17**	**7.75 ± 1.65**	**15.15 ± 2.25**
Wild	**0.56 ± 0.21**	**6.54 ± 0.91**	**13.60 ± 1.23**

Data are expressed mg/g dry weight and are mean ± standard deviation from three independent experiments (Franco et al. 2019)

## Chemical constituents

Table 3 below shows the analysis of the phytochemical constituent in the fruit of *M. communis* that contained oils, alkaloids, flavonoids, phenolics, coumarins, saponosides, tannins, quinines, and anthraquinones (Mahboubi and Ghazian 2010; Sumbul et al. 2012; Dellaoui et al. 2018).

**Table 3:** Values of chemical constituents

Phytochemicals	Values
Tannins	2.34 ± 0.07%
Total phenol	7.1 ± 0.03%
Volatile oil	0.12 ± 0.04%
Alkaloids	0.14 ± 0.01%
Fixed oil	0.72 ± 0.04%

Quantitative analysis showed that berries of *M. communis* contained tannins 2.34 ± 0.07% (Sumbul et al. 2012).

(Sumbul et al. 2012)

Table 4 shows the oil produced by *M. communis* from Iran is 0.17% from fruit, 1.3% from leaves when ripe, and 2.61% from leaves at flowering stage. During the flowering stage, oil production by *M. communis* leaves was α-pinene (3.8 - 23.0 %), 1,8-cineole (9.9 - 20.3 %), limonene (5.5 - 17.8 %), linalool (12.3 - 17.6 %) and α-terpinyl acetate (1.8 - 7.0 %). The compositions of the leaf oil at fruit ripening stage was highly similar to those of flowering, the main components: 1,8-cineole (24.0-36.1 %), α-pinene (22.1-22.5%), limonene (3.8-17.6 %), linalool (8.4-11.4 %), linalyl acetate (4.2-4.5%), α-terpinyl acetate (2.2-4.4 %), and geranyl acetate (1.2 %).

**Table 4:** Detailed chemical constituents of *M. communis*

Chemical constituent from *M. communis* fruit (Iran)	Chemical constituent from *M. communis* leaves in flowering stage (Iran)	Chemical constituent from *M. communis* leaves in ripen stage (Iran)	Chemical constituent from *M. communis* fruit (Turkey)	Chemical constituent from* M. communis* leaves essential oil concentration	Chemical constituent from *M. communis* fruits (Northern Iraq) (27).	From the northwestern Tunisia in Bni Mtir, Wild *Myrtus communis* aerial parts essential oil	Essential oils of myrtle leaf from Algeria	*Myrtus communis* oils from the Benslimane region—Morocco	Italy, *Myrtus communis* essential oil (31).
Essential oil 0.17%	Essential oil 2.6%	Essential oil 1.3%		α-pinene 308 μg/ml	Dodecane (11.39%)	*α*-terpineol (9.4 to 9.72%)	Oxygenated monoterpenes 37.47-55.23%	α-terpineol (15.5%)	α-pinene (41.6–28.9%)
α-pinene (28.6%)	α-pinene (3.8–23.0%)	α-pinene (22.1–22.5%)	Oxygenated monoterpenes (73.02–83.83%)	α-terpineol 41.73 μg/ml	Oleic acid methyl ester (21.18%)	1,8-cineole (42.58 to 51.39%)	Monoterpene hydrocarbons 36.30–51.36%	Geranyl acetate (11.64%)	α-terpineol (3.6%)
α-terpinyl acetate (5.4%)	Terpinyl acetate (1.8–7.0%)	α-terpinyl acetate (2.2–4.4%)	α-pinene (6.04–20.71%)	1-8 cinole (eucalyptol) 41.46 μg/ml	Linoleic acid methyl ester (27.19%)	Linalool (5.91 to 6.06%)	Sesquiterpene hydrocarbons 3.04–4.74%	Methyl eugenol (18.7%)	1,8-cineole (25.5–24.2%)
Linalool (2.3%)	1,8-cineole (9.9–20.3%)	Linalyl acetate (4.2–4.5%)	α-terpineol (8.40–18.43%)	Geranyl acetate 18.28. μg/ml	Octane3,5-dimethyl (16.47%)	Methyl eugenol (6.69 to 7.11%)	Oxygenated sesquiterpenes 1.89–2.86%	1,8-cineole (36.3%)	*trans*-myrtanol acetate (4.2–5.2%)
							1,8-cineole (25.46–32.12%)		Limonene (9.5–5.2%)
Linalyl acetate (3.4%)	Linalool (12.3–17.6%)	1,8-Cineole (24.0–36.1%)	1,8-cineole (29.20–31.40%)	Linalool 23.83 μg/ml	Stearic acid methyl ester (3.32%)				Linalool (11.7%)
Limonene (18.0%)	Limonene (5.5–17.8%)	Linalool (8.4–11.4%)	Geranyl acetate (3.98–7.54%)	Limonene 45.22 μg/ml	Tetradecane (6.69%)				
1,8-cineole (26.7%)		Geranyl acetate (1.2%)	Linalool (15.67–19.13%)		Palmitic acid methyl ester (6.80%)				
		Limonene (3.8–17.6%)							

From Turkey, four different *M. communis* fruit essential oils were analyzed chemically and showed oxygenated monoterpenes (73.02–83.83%) were found in excess. The major oil constituents were α-pinene (6.04–20.71%), α-terpineol (8.40–18.43%), 1,8-cineole (29.20–31.40%), geranyl acetate (3.98–7.54%), and linalool (15.67–19.13%) (Kordali et al. 2016).

From Northern Iraq, *M. communis* fruits components were dodecane (11.39%), oleic acid methyl ester (21.18%), linoleic acid methyl ester (27.19%), octane3,5-dimethyl (16.47%), stearic acid methyl ester (3.32%) tetradecane (6.69%), and palmitic acid methyl ester (6.80%) (Qader et al. 2017).

From the northwestern Tunisia in Bni Mtir, Wild *M. communis* aerial parts essential oil showed *α*-terpineol (9.45 to 9.72%), 1,8-cineole (42.58 to 51.39%), linalool (5.91 to 6.06%), and methyl eugenol (6.69 to 7.11%) were the main constituents (Messaoud et al. 2012).

Analysis of essential oils of myrtle leaf from Algeria through the use of GC-MS and GC showed oils having 51 constituents rich in oxygenated monoterpenes 37.47–55.23%, monoterpene hydrocarbons 36.30–51.36%, sesquiterpene hydrocarbons 3.04–4.74%, and oxygenated sesquiterpenes 1.89–2.86%. Their major compounds were α-pinene (30.65–44.62%) and 1,8-cineole (25.46–32.12%) (Berka-Zougali et al. 2012).

GC-MS analysis of *M. communis* oils from the Benslimane region of Morocco showed α-terpineol (15.5%), geranyl acetate (11.64%), and methyl eugenol (18.7%) in excess quantity. In comparison, Ouazzane-Morocco essential oil has abundant, rich 1,8-cineole (36.3%) (Harassi et al. 2019).

From Italy, *M. communis* essential oil contained α-pinene (41.6–28.9%), α-terpineol (3.6%), 1,8-cineole (25.5–24.2%), *trans*-myrtanol acetate (4.2–5.2%), limonene (9.5–5.2%), and linalool (11.7%), as well as the main extract (Flaminia et al. 2004).

*M. communis* var. *italica* leaf and flower essential oil composition was α-pinene (17.53% for flower and 58.05% for leaf) in excess. 1,8-cineole (32.84%) was seen in the stem extract due to excess oxygenated monoterpenes (Aidi et al. 2010).

From Aventis in France, *M. communis* essential oil analysis showed 1,8-cineole (19.6%), linalool (12.6%), and α-pinene (24.7%) (Mahmoudvand et al. 2015), though two samples of chemical profiling from northern and southern Montenegro beaches showed that monoterpenes were the main constituents (linalool, myrtenyl acetate, α-pinene, and 1,8-cineole). The samples had myrtenyl acetate (5.4–21.6%) and α-pinene (14.7–35.9%) (Mimica-Dukic et al. 2010).

*M. communis* leaves isolate includes phloroglucinols (myrtucommulones A-L), arjun olic acid, asiatic acid, 23-dihydroxyolean-12-en-28-oic acid, 3βcis-p-coumaroyloxy-2α, 23-hydroxyursolic acid, hederagenin, 3β-O-cis-p-coumaroyl-2αhydroxy-urs-12-en-28-oic acid, 3β-O-trans-p-coumaroyl maslinic acid, 3β-trans-p-coumaroyloxy-2α,24-dihydroxy-urs-12-en-28-oic acid, maslinic acid, coprozoic acid, jacoumaric acid, oleanolic acid, botulinic acid, ursolic acid (Rotstein et al. 1974; Taamalli et al. 2014b, c, a; Appendino et al. 2006; Khan et al. 2019; Liang et al. 2019; Franco et al. 2019).

Phenolics (ellagic acid, caffeic acid, ferulic acid, quinic acid, gallic acid, (−) epicatechin-3-*O*-gallate, (−) epigallocatechin-3-*O*-gallate, catechin, (−) epigallocatechin), and flavonoids (kaempferol, quercetin-3-*O*-glucoside, quercetin, quercetin-3-rutinoside, quercetin-3-O-rahmnoside myricetin, myricetin-3-O-galactoside, myricetins 3-O-alpha-L-rhamnoside, myricetins 3-O-beta-D-xyloside, myricetin 3-O-beta-D-galactoside, myricetin-3-*O*-arabinoside, myricetin-3-O-rahmnoside, hesperidin, esculin, patuletin) were *M. communis alcoholic extract of the fruits and leaf.* Hydrolysable b tannins (eugeniflorin D, eugeniflorin D_2_, oenothein B, tellimagrandins I, including tellimagrandins II) and condensed tannins, petunidin-3-O-glucoside, delphinidin-3-O-glucoside, malvidin-3-O-glucoside, cyanidin-3-O-glucoside, peonidin-3-O-glucoside, delphinidin-3-Oarabinoside, petunidin-3-O-arabinoside, and also malvidin-3-O-arabinoside, were identified in *M. communis* ( Aidi et al. 2010; Romani et al. 1999; Montoro et al. 2006; Yoshimura et al. 2008; Barboni et al. 2010; Tuberoso et al. 2010; Bouaziz et al. 2015; Bouaoudia-Madi et al. 2017).

The flavonoid contents and total phenol were abundant in the leaf extract (13.65 mg GAE/g dry weight as well as 250 mg GAE/g dry weight, respectively), and anthocyanin and tannin contents (176.50 ± 2.17 mg Cyd-3-glu /g dry weight and 220.81±1.21 mg CE /g dry weight), respectively, were abundant in the extract of the pericarp (Bouzabata et al. 2015).

The total phenolics and flavonoids were assessed in aqueous extracts, ethyl acetate, chloroform, methanol of *M. communis* leaves. Elevated total phenolic and total flavonoid contents (435.37 mg gallic acid equivalents/g dried weight also 130.75 mg quercetin equivalent/g dried weight, respectively) were seen in the ethyl acetate extract (Hosseinzadeh et al. 2011).

Different myrtle showed diversity in the total phenol contents (*M. communis* var. *italica*) parts; flower (15.70 mg GAE/g), stem (11.11 mg GAE/g), and leaf extract (33.67 mg GAE/g) extracts. The total tannin contents of myrtle different parts is as follows: (3.33 mg GAE/g in stem, 11.95 mg GAE/g in the flower, and 26.55 mg GAE/g in leaf). The stem extract showed the main contents of condensed tannins, total flavonoids, (5.17 and 1.99 mg CE/g individually), and leaf extract (3 and 1.22 mg CE/g, respectively). The HPLC analysis showed hydrolysable tannins (gallotannins) in leaf (79.39%, 8.90 mg/g) and flower (60.00%, 3.50 mg/g) are the predominant class of phenolics, whereas the flavonoid class is the most abundant (61.38%, 1.86 mg/g) due to excess catechin (36.91%, 1.12 mg/g) (Mimica-Dukic et al. 2010).

The phenolic compounds content in myrtle aerial parts infusions with different times of preparation ( 5, 10 and 15 min), respectively, was (μ mol/g dry matter): gallic acid 6.47 ± 0.59, 8.23 ± 1.23, and 11.82 ± 0.65; caffeic acid 0.71 ± 0.18, 0.88 ± 0.29, and 1.41 ± 0.06; syringic acid 0.18 ± 0.06, 0.29 ± 0.24, and 0.53 ± 0.12; ferulic acid 0.29 ± 0.08, 0.41 ± 0.06, and 0.53 ± 0.13; myricetin-3-ogalactoside 0.59 ± 0.09, 0.76 ± 0.08, and 1.06 ± 0.15; myricetin-3-orhamnoside 0.71 ± 0.11, 0.82 ± 0.10, and 1.18 ± 0.16; myricetine-3-oarabinoside 0.12 ± 0.09, 0.18 ± 0.07, and 0.24 ± 0.04; quercetin-3-O-galactoside 5.35 ± 0.18, 6.64 ± 1.29, and 9.11 ± 1.00; quercetin-3-O-rhamnoside 0.29 ± 0.18, 0.29 ± 0.12, and 0.53 ± 0.17; myricetin 3.00 ± 0.24, 3.82 ± 0.29, and 5.00 ± 0.65; quercetin 1.59 ± 0.47, 2.41 ± 0.35, and 3.59 ± 0.59; total identified phenolic compounds 19.28 ± 2.12, 24.75 ± 4.06, and 34.98 ± 3.47; phenolic acids 7.64 ± 0.88, 9.82 ± 1.82, and 14.28 ± 0.88; flavonol glycosides 7.05 ± 0.53, 8.70 ± 1.59, and 12.11 ± 1.35; and flavonols 4.58 ± 0.71, 6.23 ± 0.65, and 8.58 ± 1.23 (Harassi et al. 2019).

Mineral contents of the leaves of *M. communis* were: P 547± 28, Na 133 ±19, Zn 25 ±1, Si 2 ±3, Cu 9.1 ±1, Ni 0.5 ±0.1, Cr 2±0.4, Pb 0.5 ±0.5, Mo 0.5 ±0.1, V 0.27 ± 0.1, and Cd 0.24 ±0.1 µg/g dry weight (Messaoud et al. 2012).

## Pharmacological effects

**Fig. 2:**
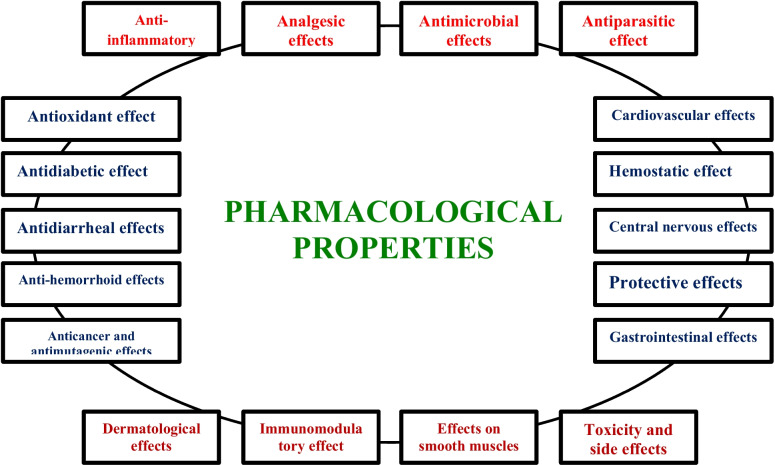
Schematic diagram for the pharmacological properties of *M. communis*

The pharmacological effects of *M. communis* are summarized in Fig. 2 and Table 5 below.

**Table 5:** Overview of the pharmacological effects of *M. communis.*

Pharmacological effects	Activity model	Plant part used	References
Anti-inflammatory	Lipopolysaccharide (LPS)-stimulated macrophages in vitro model	Seeds	Cruciani et al. 2019, Soomro et al. 2019, Dragomanova et al. 2019, Alem et al. 2008, Maxia et al. 2011, Rossi et al. 2009, Raoof et al. 2019.
Analgesic effects	Mouse model	Aerial part	Koeberle et al. 2009
Antimicrobial effects	*P. aeruginosa*,* S. shigie*,* S. aureus*,* S. typhi*,* E. coli*,* K. aerogenes*,* P. vulgaris*, and* P. mirabilis*	Leaves, seeds	Barboni et al. 2010, Feuillolay et al. 2016, Teimoory et al. 2013, Rasaie et al. 2017, Appendino et al. 2006, Harassi et al. 2019, Rahimvand et al. 2018, Fani et al. 2014, Ben et al. 2014, Javadi et al. 2017, Cruciani et al. 2019, Beni et al. 2017, Flaminia et al. 2004, Cannas et al. 2013, Mert et al. 2008, Aleksic et al. 2014, Fadil et al. 2018, Masoudi et al. 2017, Cannas et al. 2014,Barac et al. 2018, Mahdi et al. 2006, Mehrabani et al. 2013, Penauelas et al. 2001
Antidiabetic effects	Mouse model	Berry, leaves	Baz et al. 2016, Panjeshahin et al. 2016, Karimlar et al. 2019, Tas et al. 2018, Medhat et al. 2017, Al-Jeboory et al. 1985, Taamalli et al. 2014b, c, a
Antiparasitic effects	*Trogoderma granarium* model	Leaves	Chegeni et al. 2019, Amiri et al. 2019, Tayoub et al. 2012, Amensour et al. 2009, Dairi et al. 2014, Shahnazi et al. 2017, Rotsein et al. 1974
Antioxidant effects	Different models were used such as K562 cell line, ABTS assay, ORAC assay	Leaves, berry	Tumen et al. 2012, Ines et al. 2012, Wannes and Marzouk 2016, Rosa et al. 2003, Demir et al. 2016, Elfellah et al. 1984, Rosa et al. 2008, Mimica-Dukic et al. 2010, Cottiglia et al. 2012, Flaminia et al. 2004
Cardiovascular effects	Guinea pig, rabbit and rat model	Leaves	Ebrahimi et al. 2020, Hosseinzadeh et al. 2011, Youness et al. 2016
Hemostatic effects	Rat model	Leaves	Hajiaghaee et al. 2016
Central nervous effects	Mouse and rat model	Leaves, berry	Romani et al. 2004, Shahmohammadi et al. 2012, Aykac et al. 2019, EL-Kholy et al 2018, Naji et al. 2018
Protective effects	Rat model	Leaves	Sen et al. 2016, Samareh e al., 2018, Ozbeyli et al. 2020, Mahboubi et al. 2016, Zohalinezhad et al. 2016
Gastrointestinal effects	Rat model	Berry	Jabri et al. 2016a, Bouzabata 2013, Benchikh et al. 2016, Sumbul et al. 2012
Antidiarrheal effects	Rat model	Berry, Seeds	Sisay et al. 2017, Panahi et al. 2014, Mahboubi et al. 2017
Effects on smooth muscles	Rabbit model	Leaves	Ozturk and Duran 2018
Anti-hemorrhoid effects	Patients	Seeds	Malekuti et al. 2019, Janbaz et al. 2013, Harassi et al. 2019
Anticancer and antimutagenic effects	Different models were used such as Vero cells and HCT 116 cell model, MCF-7 and P815 cells, brine shrimp, *Escherichia coli*	Leaves	Tretiakova et al. 2008, Fani et al. 2014, Hayder et al. 2008, Maxia et al. 2011, Choudhary et al. 2013, Ghadami et al. 2014, Cottiglia et al. 2012
Immunomodulatory effect	Myrtucom muacetalone and growth regulator G3 factor	Leaves	Ozcan et al. 2019
Dermatological effects	Rat model	Leaves	Salehpour et al. 2016, Hayder et al. 2008
Toxicity and side effects	Sheep model, mouse model	Aerial part, leaves	Soomro et al. 2019, D’Urso et al. 2017

## Anti-inflammatory

*M. communis* essential oil was assessed for anti-inflammatory property using lipopolysaccharide (LPS)-stimulated macrophages in vitro model; oils significantly inhibited the production of NO without altering the viability of the cell at 0.64 mg/ml concentrations (Cruciani et al. 2019). The aqueous extracts (0.03, 0.015, 0.005 g/kg) and ethanolic (0.05 g/kg) possessed anti-inflammatory action against prolonged inflammation (Soomro et al. 2019). *M. communis* essential oil anti-inflammatory activity was assessed by mice with ear edema induced by croton oil and myeloperoxidase activity, and in rats’ models, granuloma induced by cotton pellet and serum TNF-α as well as IL-6 (Feisst et al. 2005). The topical administration of the essential oil reduced edema in the ear, cotton pellet-induced granuloma, MPO activity, serum IL-6, and TNF-α. The bicyclic monoterpenoid myrtenal, which was isolated from *M. communis* essential oil (5%), was tested for anti-inflammatory effects in rodent models.

The anti-inflammatory action of oenothein B isolated from *M. communis* seeds was assayed in vitro. It possessed anti-inflammatory properties.* M. communis* pulp and seed extracts were evaluated on human fibroblast for their effects against inflammation. The production of ROS was determined after H_2_O_2_ treatment induced oxidative stress and expressions of gene of various proinflammatory cytokines and CYP3A4 and CYP27B1. The results revealed the synergic effect of *Myrtus* extracts with vitamin D to reduce inflammation, ROS production, thereby shielding cells from damages caused by oxidative stress, modulated CYP expression, and preventing chronic inflammation (Dragomanova et al. 2019).

*M. communis* leaf isolate oligomeric non-prenylated acylphloroglucinols (semi myrtucommulone and myrtucommulone A) directly inhibited 5-lipoxygenase and COX-1 in vivo, including in vitro, with IC_50_ value around 1.8 to 29 microM, thereby significantly reducing eicosanoids biosynthesis. In polymorphonuclear leukocytes, calcium mobilization is prevented by the administration of myrtucommulone and semi myrtucommulone, which suppresses the concentration ROS and elastase formation, which is mediated through the signaling pathway of G protein of IC_50_ values around 0.55 and 4.5 microM, respectively (Alem et al. 2008).

*M. communis* extract myrtucommuacetalone-1 (MCA-1) was assessed for anti-inflammation using hydrogen peroxide, superoxide, and macrophages nitric oxide assays. The translocation and phosphorylation transcription factor NF kappa B, iNOS activation regulator, was used to analyze the compound. MCA-1 action on the iNOS enzyme was also tested. In excited macrophages, MCA-1 caused the inhibition of hydrogen peroxide, superoxide, with 53% and 48% respectively, through nitric oxide an IC_50_ of <1 µg/ml, showing solid binding pattern at the enzyme nitric oxide synthase active site. In addition, MCA-1 of 25 µg/ml stopped the expression of inducible nitric oxide synthase and eliminated the translocation and phosphorylation of transcription factor (NFκB) into the nucleus (Maxia et al. 2011).

*M. communis* extract acylphloroglucinol myrtucommulone effect on PGE2 synthase mPGES-1 and subsequent inhibition of prostaglandins as an intermediary of pain and inflammation was examined using the A549 cells stimulated by interleukin-1beta- prepared in a microsomal means for a precursor for mPGES-1, lipopolysaccharide-stimulated human whole blood, and intact cells A549 in a cell-free assay. Production of 6-oxo PGF 1α and 12(S)-hydroxy-5-cis-8,10-trans-heptadecatrienoic acid confirmed that the activity of COX-1 and COX-2 was inhibited in the cell-free and cellular tests. The conversion of PGE2 from PGH2 was done by cell-free mPGES-1-mediated (IC_50_ = 1 micromole/l). Myrtucommulone concentration-dependently inhibited the production of PGE2, which decreased significantly in whole human blood and complete A549 cells at small micromolar concentrations. Activities of COX-2 in A549 cells and isolated human recombinant COX-2 were significantly inhibited, but COX-1 was inhibited slightly in cell-free and cells by the action of myrtucommulone (30 micromole/l) and (IC_50_> 15 micromole/l) respectively (Rossi et al. 2009).

Using a mouse model, inflammation was brought about by the intrapleural administration of carrageenan, while intraperitoneal of treatment myrtucommulone was given (0.5, 1.5, and 4.5 mg/kg). The result obtained showed the suppression of proinflammatory cellular response. The administration of 4.5 mg/kg myrtucommulone ip at half of an hour before and after the introduction of carrageenan causes the decrease in the amount of leukocyte in the blood, neutrophil infiltration seen through myeloperoxidase, lung injury, lung intercellular adhesion molecule-1 including P-selectin immunohistochemical localization, an equally pleural fluid reduction in the cytokine levels (TNF-α and IL-1β) of the lungs, leukotriene B4. In the pleural fluid, and their immunohistochemical localization in the lung, the leukotriene B4, but not prostaglandin E2, levels in the pleural exudates and lung peroxidation (thiobarbituric acid-reactant substance) and nitrotyrosine and poly (ADP-ribose) immunostaining. Myrtucommulone reportedly suppressed eicosanoids biosynthesis by inhibiting cyclooxygenase-1 and 5-lipoxygenase in vitro, ROS, and elastase formation in activated polymorphonuclear leukocytes (Raoof et al. 2019).

## Analgesic effects

*M. communis* aerial parts of both aqueous and ethanolic extracts were tested for antinociceptive activity by testing with a hot plate and writhing. Antinociception was shown by the aqueous and ethanolic extracts, with marked antinociception inhibited through naloxone. Antinociception was exhibited by extracts against the writhing induced by acetic acid.

The bicyclic monoterpenoid myrtenal, which was isolated from *M. communis* essential oil (5%), was tested for analgesic. Anti-nociceptive activity (30 mg/kg, bw, ip) was tried on two experimental pain models of male mice using a writhing test of acetic acid including the hot plate, after single administration in addition to repetitive administration.

The bicyclic monoterpenoid myrtenal showed significant antinociception (concerning thermal and peripheral pain). During severe administration, the writhing number of the abdomen significantly decreased in the 15^th^ and 20^th^ minutes (at *P*< 0.01 and *P*< 0.05) by 47.25% and 50.55% respectively. It reduced (*P*<0.001) treatment group number versus control group after continuous treatment, 40.4% on 7^th^ and 14^th^ day 43.1% when assessed with the controls (Koeberle et al. 2009).

## Antimicrobial effects

*M. communis* seed isolate oenothein B was tested for its fungicidal effect on *Candida *in vitro, and its MIC anticandidal effect was <8–64 μg/ml (Barboni e al. 2010).

The crude extract of myrtle was examined for antimicrobial action against *Pseudomonas aeruginosa*, *Staphylococcus aureus*, *Salmonella typhi*, *Escherichia coli*, *Klebsiella aerogenes*, *Proteus vulgaris*, and *Proteus mirabilis*. The MIC of the extract was (0.5, 2.5, 15, and 20 mg/ml) against *S. aureus*,* P. mirabilis*, *P. vulgaris*,* Klebsiella*,* S. typhi*, and *P. aeruginosa*, respectively. However, there was 18-folds increase in the antibacterial activity after autoclaving at 121 °C for 15 min (Feuillolay et al. 2016).

*M. communis* aqueous extract possessed antibacterial properties against *Escherichia coli* (zone of inhibition 11.48–13.04 mm at 50 mg/ml),* Bacillus subtilis* (zone of inhibition 10.80–13.72 mm at 50 mg/ml), and *Pseudomonas aeruginosa* (zone of inhibition 14.06–18.76 mm at 50 mg/ml), but did not show fungicidal against *Aspergillus oryzae* (Teimoory et al. 2013).

*M. communis* leaf ethanolic extract was examined for antibacterial effect (0, 15, 30, 45, 60 mg/ml) against Gram-positive bacteria (*Bacillus subtilis* and *Staphylococcus aureus* and Gram-negative bacteria (*Escherichia coli* and* Klebsiella pneumonia*)*.* The results revealed the potency of extracts against all the bacteria tested. *Escherichia coli* showed the highest sensitivity to the extract of *M. communis*. The extract was applied in the study of its action on biofilm formation. High sensitivity was seen when biofilm formation was assessed on all the bacterial strains with *Escherichia coli* and* Bacillus subtilis* being the most potent to *M. communis* extract (Sidkey 2006; Rasaie et al. 2017). An acylphloroglucinols (myrtucommulone-A) isolated from *M. communis* leaves exhibited great antibacterial activity versus Gram-positive bacteria, though inactive on the Gram negatives (Appendino et al. 2006).

The antimicrobial effect of *M. communis* extracts was studied against* E. coli*, *E. coli* ATCC 25922, *P. mirabilis*, *S. typhi*, *Shigella flexneri*, and* K. pneumonia.* All *M. communis* inoculates exhibited various degree of inhibition antimicrobial activity against all strains tested. The microbial extracts and strains showed inhibition zones values ranging from 21 to 25 mm. According to microbial strains and infusions. The values of MBC and MIC showed ranges from 12.5 to 25 mg dry leaves/ml and 12.5 to 50 mg dry leaves/ml against different isolates, respectively (Harassi et al. 2019).

*M. communis* alcoholic extract was assessed for its bactericidal effect against *Listeria monocytogenes*, *Bacillus cereus* (NCTC 7464 and ATCC 10876), and *Staphylococcus aureus* (ATCC 25923). At a concentration of 10 mg, *M. communis* demonstrated a growth inhibitory effect on the three examined bacteria. A maximum effect was observed on *Staphylococcus aureus* and a minimum effect against *Bacillus cereus* (Rahimvand et al. 2018)*. M. communis* leaf extracts of water extracts, methanol, n-hexane, ethanol, and ethyl acetate were examined for its antimicrobial activities against *Candida albicans* ATCC 10239, *Escherichia coli* ATCC 25922, *Escherichia coli* ATCC 29998, *Escherichia coli* ATCC 11230, *Staphylococcus aureus* ATCC 6538P, *Staphylococcus aureus* ATCC 29213, *Staphylococcus epidermidis* ATCC 12228, *Enterococcus faecalis* ATCC 29212, *Enterobacter cloacae* ATCC 13047, *Pseudomonas aeruginosa* ATCC 27853, and *Salmonella typhimurium* CCM 5445. The growth of *Salmonella typhimurium*, *Pseudomonas aeruginosa*, *Escherichia coli*, *Staphylococcus epidermidis* was stopped by all the extracts. The methanolic extract was potent against the growth of *Escherichia coli.* All the extracts did not show any activity against *Enterococcus faecalis* ATCC 29212, *Candida albicans*, or *Enterobacter cloacae* ATCC 13047 (Fani et al. 2014).

*M. communis* methanolic and aqueous extract antibacterial effect was examined against* Prevotella intermedia*,* Porphyromonas gingivalis*, and *Actinobacillus actinomycetemcomitans*. *M. communis* aqueous extract with a range 20 to 500 mg/ml and methanolic extract with a range 10–500 mg/ml possessed antibacterial effects against *Prevotella intermedia*,* Porphyromonas gingivalis*, and *Actinobacillus actinomycetemcomitans*. *M. communis* aqueous as well as methanolic extracts showed potency against microorganisms examined with a MIC of 10 mg/ml (Ben et al. 2014).

Ethyl acetate, total methanolic extracts, and extracts of* M. communis* exhibited good fungicidal action against *Microsporum canis*, *Microsporum gypseum*,* Trichophyton mentagrophytes* (Javadi et al. 2017); also, the essential oil acted against *Epidermophyton floccosum*,* Cryptococcus neoformans*,* Trichophyton rubrum*, and *Microsporum canis* was assessed, respectively (Cruciani et al. 2019). *M. communis* essential oils exhibited exceptional antimicrobial activities against *Staphylococcus aureus*,* Escherichia coli*, and* Candida albicans. M. communis* oils exposure showed the D-values (decimal reduction value) of (12.8, 2.8, and 8.6 min) *S. aureus*, *E. coli*, and *C. albicans*, respectively (Yadegarinia et al. 2006; Beni et al. 2017). 

Myrtle leaf essential oil was extracted using conventional hydro distillation including solvent-free-microwave and the antimicrobial activity against Gram-positive bacteria (*Bacillus subtilis*, *Listeria monocytogenes*,* Staphylococcus aureus*) Gram-negative bacteria (*Salmonella enteric*, *Escherichia coli*, *Enterobacter cloacae*, *Klebsiella pneumonia*,* Pseudomonas aeruginosa*), and fungi and yeast (*Aspergillus flavus*, *Candida albicans*, *Aspergillus ochraceus*, and* Fusarium culmorum*). Both essential oils exhibited high microbicidal action against both Gram-positive and Gram-negative bacteria (MIC 10–30 µl/ml), yeast, and fungi (MIC 10–50 µl/ml) (Flaminia et al. 2004).

*M. communis* essential oil microbicidal effect was examined against *Streptococcus mutans*, *Streptococcus sanguinis*, and *Streptococcus salivarius*. According to the results, myrtle leaf essential oil possessed microbicidal activity against every strain of *Streptococcus*; however, *S. mutants* has the most of all the species (Cannas et al. 2013).

*M. communis* oil antimycotic activity was assessed against *Candida* spp. from clinical isolates. The MIC ranges obtained with the essential oil of myrtle after 24 h were 0.5–2 μg/ml, 0.25–1 μg/ml, 1–4 μg/ml, 0.5–4 μg/ml, 0.5–2 μg/ml for *Candida parapsilosis*, *Candida tropicalis*, *Candida krusei*, *Candida glabrata*, and* C. albicans*, respectively*.* The ranges of MIC after 48 h include 0.5–4 μg/ml, 0.5–2 μg/ml, 2–4 μg/ml, 1–4 μg/ml, 1–2 μg/ml for *C. parapsilosis*,* C. tropicalis*,* C. krusei*, *C. glabrata*, and *C. albicans*, respectively (Mert et al. 2008).

The *M. communis* essential oil antimicrobial activities (3.9–1000 µg/ml) were studied against some oral pathogens (30 strains of *Aggregatibacter actinomycetemcomitans*,* Porphyromonas gingivalis*, and *Streptococcus mutans* and 20 strains of *Candida albicans* and *Streptococcus pyogenes*) gotten out of pharyngitis, periodontal diseases, dental caries, and oral lesions related to artificial dentures. All the extracts had 8.1– 41.25-mm inhibition zones, with the sensitivity to oil at 125–1000 µg/ml. At 62.5 µg/ml, the strain sensitivity of *C. albicans*, *S. mutans*, and *S. pyogenes* was recorded, but only 70% and 66.6% of *A. actinomycetemcomitans* and* P. gingivalis* showed resistance to this concentration*.* At 31.25 µg/ml, the sensitivity of all the strains,* S. mutans*, and* S. pyogenes* was recorded*.* The oil at 7.8 and 15.6 µg/ml sensitivity of *S. pyogenes* strain was recorded. At 62.5 ± 0, 62.5 ± 0, 46.9 ± 16, 31.25 ± 0, 29.68 ± 4.8 µg/ml, the minimum inhibitory concentrations were recorded against the oil for these microorganisms: *P. gingivalis*,* A. actinomycetemcomitans*,* C. albicans*,* S. mutans*, and *S. pyogenes*, respectively (Aleksic et al. 2014)*.*

Some microorganisms were able to access microbicidal activity of essential oils. The ranges for MIC and the zones of inhibition and of the bacteria strains are 0.078–2.5 mg/ml and 16–28 mm, respectively. Gram-negative bacteria showed lower oil activity of inhibition than the gram-positive ones. The oil inhibited several fungal strains significantly. The oil concentration at 312 μg/ml showed bactericidal action against* Listeria monocytogenes* after 5 min (Fadil et al. 2018).

Three essential oils of *M. communis* were examined for their action against *Acinetobacter baumannii* extracted from wound known to be multi-drug. Mytre oils exhibited excellent bactericidal effects, as shown by the result. The drug ciprofloxacin or polymyxin B, when combined with essential oil sun inhibition concentration, decreased the growth of bacterial interdependently (Masoudi et al. 2017).

A synergistic effect was noticed against the strain of *Salmonella typhimurium* by combining *Thymus vulgaris* and *M. communis* essential oils. The preparations include 45% of *M. communis and* 55% of *Thymus vulgaris* essential oil, which improved the sensitivity of *Salmonella typhimurium* (Cannas et al. 2014).

For bacterial vaginosis treatment, *M. communis* vaginal gel was used alongside with metronidazole gel for 7 days. It was discovered that the administration showed higher potency (*P*<0.05) compared to their use when not in combination. After 21 days, the patients that were treated with only metronidazole gel had bacterial vaginosis reoccurring, but others administered with a combination of *M. communis* in metronidazole gel did not show any relapse of the disease (Barac et al. 2018).

The essential oil of *M. communis* was examined for the formation of biofilm examined in three *Candida* species (*Candida tropicalis*,* Candida albicans*, and* Candida parapsilosis*). The results showed enhanced action against *C. parapsilosis* and *C. albicans* as compared to* C. tropicalis*. Therefore, anti-biofilm formation is one of its activities (Mehrabani et al. 2013).

The essential oil of *M. communis* was examined by isolating Malassezia sp. In pityriasis versicolor infection, fungicidal and inhibitory action was seen against *Malassezia* growth (Mahdi et al. 2006).

Its fungicidal action was examined using *M*. *communis* essential oil on various *Aspergillus* sp. (six isolates of *Aspergillus flavus*, *Aspergillus parasiticus*, and *Aspergillus niger*) and* Candida albicans* (one ATCC type strains and eight clinical isolates), which showed good inhibitory action against these strains. Also, the combination of amphotericin and essential oil showed an excellent synergistic effect (Penauelas et al. 2001).

## Antiparasitic effect

At pH 4.65, *M. communis* extract killed *Trichomonas vaginalis*, but at pH 6.00, it was still alive (Chegeni et al. 2019). Both cell culture and cell-free medium were used in anti-*Toxoplasma* action on *M. communis* extracts in vitro against *Toxoplasma gondii* RH strain tachyzoites. The extract caused the inhibition of tachyzoites in cell-free medium. However, the cell culture of *M. communis* extract and EC_50_ sensitivity were significantly decreased compared to pyrimethamine (Amiri et al. 2019). At 6.47, 7.62, and 11.64 mg/ml, the LC_50_ was recorded at 30, 20, and 10 min, respectively (Tayoub et al. 2012). Apoptosis of protocolizes of hydatid cyst was seen when *M. communis* extract at (50 and 100 mg/ml) was administered through the increased action of caspases 3 and 9 (Amensour et al. 2009).

The essential of *M. communis* leaf extract possesses fumigant toxicity against *Trogoderma granarium* at different stages. At 24 and 48 h of exposure to 562.5 µl/l air, the larva showed 94% and 100% death, but the adult was very sensitive at little exposure. The LC_90_ and LC_50_ include 487 and 221 µl/l air after 48-h exposure, respectively (Dairi et al. 2014).

In vitro analysis of *M. communis* extract in water and ethanol (10, 5, 2.5, and 1.25 mg/ml) was conducted for the anti-*Acanthamoeba* effect of crude for (1–3 day) cysts of *Acanthamoeba* and on trophozoites. After administration, *M. communis* ethanolic extract showed 0% for trophozoites viability and 8.62% for cysts.

After 3 days, 0% trophozoites and 31.10% cysts were seen after administering *M. communis* aqueous extract at 10 mg/ml (Shahnazi et al. 2017). *M. communis* methanolic and essential oil extracts had a leishmanialicidal effect on the amastigote and promastigote types of* Leishmania tropica*. The essential oil of *M. communis* brought about apoptosis to the J774 cells (*P*<0.05), amastigote and promastigote types of *L. tropica* in a manner that is dependent on dose. The amastigote as well as promastigote forms showed 11.6 and 40.8 μg/ml and 8.4 and 28.9 μg/ml as their IC_50_ values after the essential oil and methanolic extract administration. The extract of methanol and essential oils was not cytotoxic to J774 cells. It was seen that *M. communis* essential oil was more cytotoxic than the methanolic extract (Rotsein et al. 1974). *M. communis* methanolic extract administered against hydatid cyst protoscoleces showed scolicidal effect at 50 and 100 mg/ml concentrations.

## Antioxidant effect

The assays of reducing antioxidant power, and beta-carotene linoleic acid were used to examine action against oxidants, leaf total phenolic content, and *M. communis* berries. The ranges of the total phenol content include 9.0 mg and 35.6 mg GAE/g of myrtle extract. The berry extracts possess fewer total phenolic compounds, but the leaves have more total phenolic compounds. The aqueous, ethanol, methanol of both leaves and berry extract showed antioxidant activity, but these activities were higher in leaves. The order of these activities includes berry extract (methanol > ethanol > water) and leaf extract (methanol > water > ethanol), respectively (Tumen et al. 2012). The antioxidant activities of phenolic compounds extracted by conventional hydro distillation and microwave-assisted extraction were investigated using different models. The extracts produced by the two extraction techniques produced phenolic compound content that were alike. According to ABTS assay, the extract of myrtle was the highest scavenger compared to α-tocopherol and butylated hydroxyanisole. In ORAC assay, butylated hydroxytoluene was not as strong as both extracts, but the strongest were as myricitrin (myricetin 3-O-rhamnoside) and caffeic acid. The manufacture of conjugated diene andCu^2+^-induced LDL oxidation during the lag phase responded in a manner dependent on the dose, as myrtle extract and myricitrin inhibited them all. Micelles appearance and bilayer vesicles as seen in the dispersion phospholipid analysis of bile salts through cryo-electron microscopy in bile salts oxidation or 2,2′-azobis (2-amidinopropane) hydrochloride-induced phospholipid myricitrin exhibited a greater protective effect than myrtle extracts, despite the similar effects of caffeic acid with tocopherol and myrtle extract (Ines et al. 2012). *M. communis* leaf extract 5-O-di-galloylquinic acid (DGQA), when administered on K562 cell line, inhibited lipid peroxidation induced by H_2_O_2,_ pointing out its antioxidant activity. Also, 82.2% of malondialdehyde formed was inhibited by the pure sample, which equally inhibited H_2_O_2_-induced genotoxicity. DGQA increased DNA repair enzymes and antioxidant enzymes; it also prevented gene expression in H_2_O_2_ stressed chronic myelogenous leukemia cell line (K562) (Wannes and Marzouk 2016). *M. communis* extracts showed high antioxidant activity due to derivatives of gallonyl and polyphenols present.

The composition of the hydroalcoholic extracts is ellagitannins, flavanol glycosides, galloyl-quinic acids, galloyl-glucosides, aqueous residues, and ethyl acetate extracts, which have excess flavanol glycosides and hydrolysable tannins (galloyl-quinic acids, ellagitannins, galloyl-glucosides) (Rosa et al. 2003).

The unique oligomeric, nonprenylated acylphloroglucinols (semi myrtucommulone and myrtucommulone A) are* M. communis* leaf isolate with great antioxidant properties, protected linoleic acid action against free radical attack, FeCl_3_^−^ and autoxidation inhibition, including EDTA-mediated oxidation. Although the two compounds lack pro-oxidant action, semi myrtucommulone is more potent than myrtucommulone A. It was seen more in hepatic cells homogenates for action against ferric-nitrilotriacetate-induced lipid peroxidation than in cytotoxicity cell cultures for TBH inhibition or oxidation of induced FeCl_3_. The overall effects confirmed that semi myrtucommulone was a novel dietary antioxidant lead (Demir et al. 2016).

The exceptional oligomeric non-prenylated acylphloroglucinols (myrtucommulone A and semi myrtucommulone) displayed great antioxidant properties in solvent-free and cholesterol thermal (140 °C) degradation. There was significant preservation of LDL from oxidative damage of induced Cu^2+^ ions after 120 min of oxidation due to the semi myrtucommulone and myrtucommulone. In pre-treatment, oxidative products (7-ketocholesterol, 7-beta-hydroxycholesterol, and conjugated dienes fatty acids hydroperoxides) were inhibited, pointing out great protection because of reduced cholesterol and polyunsaturated fatty acids (Elfellah et al. 1984).

The antioxidant effect of the methanolic extract of the seeds and oil was assessed using 1,1-diphenyl-2-picrylhydrazyl radical scavenging, reducing power β-carotene-linoleic acid bleaching, and metal chelating activity assays. In all tests of myrtle seed methanolic extract, the methanolic extract of myrtle seed exhibited enhanced antioxidant action to the oil (Rosa et al. 2008).

The essential oil and methanolic extract of *M. communis* var. italica variety were examined for their antioxidant activity using assays like beta-carotene-linoleic acid bleaching, metal chelating activity, and reducing power assays. The methanolic extracts of various parts of myrtle in all the tests displayed more antioxidant action compared to the essential oils (Mimica-Dukic et al. 2010; Cottiglia et al. 2012).

Antioxidant activity was exhibited by the essential oil of Myrtle leaf extracted by solvent-free-microwave extraction and conventional hydro distillation; however, their antioxidant effects were significantly lesser than the quercetin and gallic acid extract. The essential oil of fresh myrtle leaf obtained by solvent-free-microwave extraction showed more antioxidant activity than the one found in conventional hydro distillation (Flaminia et al. 2004).

## Antidiabetic effect

*M. communis* berry aqueous extract was assessed for its hepatoprotective and antidiabetic effects (oral administration for 14 days with 250, 500, or 1000 mg/kg) in diabetic rats induced with streptozotocin. The extract administration significantly reduced AST, ALT, ALP, and serum glucose levels in all the diabetic groups. For the antioxidant, level of activity of SOD and GSH significantly increased, while a reduction of MDA was noticed in the diabetic rats as compared to control (*P*<0.05). The antioxidant and hypoglycemic effect was seen at a dose of 1000 mg/kg (Baz et al. 2016). *M. communis* aqueous with an ethanolic extract of (2 g/kg) when intragastrically administered 30 min prior to the injection of streptozotocin eliminated the early hyperglycemia. *M. communis* extract given 24 h and 30 h prior to streptozotocin inhibited the development of hyperglycemia 48 h later. Hyperglycemia was reduced significantly after administering Myrtus extract 48 h after streptozotocin; this effect was seen after administration. In normal mice, the blood glucose level was unaltered upon Myrtus extract administration (Panjeshahin et al. 2016).

*M. communis* leaf aqueous extract was investigated for antidiabetic and antioxidant effects in diabetic rats induced with streptozotocin (STZ) and normal rats. MC leaf aqueous extract given at 150, 300, and 600 mg/kg significantly reduced ALT, AST, ALP in serum blood glucose and MDA levels, in STZ-induced diabetic rats when compared to controls groups (*P*<0.05) (Karimlar et al. 2019).

The leaf hydroalcoholic extract displayed a mild antidiabetic effect in streptozotocin induced diabetic rats. However, the ethanolic extract of leaves (2 g/kg) possessed superior hypoglycemic effect in the diabetic rats than the aqueous extract (*P* < 0.05) (Tas et al. 2018). The hypolipidemic and hypoglycemic effects of *M. communis* hydroalcoholic fruits extract were studied in diabetic rats induced with streptozotocin. *M. communis* hydroalcoholic fruits extract was supplemented with drinking water for 35 days. *M. communis* hydroalcoholic fruit extract group exhibited reduced lipid profile, serum glucose, and level of malondialdehyde in tissue but increased erythrocyte SOD, aryl esterase, total blood GSH-Px, insulin, and serum paraoxonase activities (Medhat et al. 2017).

Studies were made on lipid profile and blood glucose in diabetic rats induced with streptozotocin-treated *M. communis* essential oil (200 mg/kg/day). Results pointed out its ability to inhibit α-glucosidase activity in vitro. However, triglyceride (TG), total cholesterol (TC), serum glucose, and low-density lipoprotein cholesterol (LDL) were significantly elevated in diabetic rats than control group (*P*<0.001). For the normal and diabetic control group, high-density lipoprotein cholesterol (HDL) did not show significance. *M. communis* oil causes a significant decrease in TC (107±11 and 83±13, *P*<0.01), TG (167±13 and 118±13, *P*<0.001), LDL (70±8 and 47±4, *P*<0.001), glucose (478±24 and 355±48, *P*<0.001), and increased HDL (37±5 and 53±9, *P*<0.01). The oil also significantly inhibited α-glucosidase activity (Al-Jeboory et al. 1985).

*M. communis* possessed α-glucosidase inhibitory activity and protein tyrosine phosphatase 1B (PTP1B), with IC_50_ values ranges from 34.3 to 88.5 μM and 8.9 to 69.4 μM, respectively. The results supported *M. communis* application as a bifunctional food for type-2-diabetes treatment or management (Taamalli et al. 2014b, c, a).

## Cardiovascular effects

*M. communis* aqueous extract showed an adverse inotropic effect on isolated Guinea pig atria, which atropine administration did not reverse. The whole extract induced cardiac depressive concentration-dependent effect in rabbits under an anaesthesia (Ebrahimi et al. 2020).

The hypotensive effects of *M. communis* methanolic and ethyl acetate leaf extracts were assessed in rats under an aesthesia by using invasive blood pressure recording. Intravenous administration of methanol and ethyl acetate reduced the maximum mean arterial systolic and diastolic blood pressure by 32.49% and 20.6% at 12 mg/kg bw, respectively, in rats anesthetized (Hosseinzadeh et al. 2011).

Studies were done to assess the leaf extract of *M. communis* ability to mitigate endothelial dysfunction and atherosclerosis in ovariectomized rats to model post menopause. Parameters assessed were antioxidant, aortic oxidant, asymmetric dimethyl arginine, interleukin 1beta, lipid profile, plasma estrogen, von Willebrand factor, lipoxin A4, and erythrocyte membrane fatty acids. The inflammatory and oxidant parameters were significantly increased in ovariectomized rats, but the administration of *M. communis* extract (100 mg/kg bw) for about 60 days reduced the treated group values. The leaf extract of *M. communis* possessed protective effects against endothelial dysfunction and atherosclerosis in ovariectomized rats because of its high amount of antioxidant and anti-inflammatory compounds, including ω-3 fatty acids (Youness et al. 2016).

## Hemostatic effect

The application of 5% extract to the cut tail in the bleeding model of rat-tail significantly reduced the time of bleeding (*P*< 0.001) as compared to normal saline group. The figures of PT and aPTT were elevated >120 s and >180 s by 5% extract, respectively. Likewise, serum proteins and protein precipitation were significantly reduced in the extract group of extract with 5% (Hajiaghaee et al. 2016).

## Central nervous effects

The acetone, dichloromethane, ethyl acetate, and methanol extracts (200 μg/ml) of the leaf and berry extracts of *M. communis* were screened beside butyrylcholinesterase, acetylcholinesterase, and tyrosinase. All these enzymes are linked with neurodegenerative diseases. The extracts exhibited a mild acetylcholinesterase (17.49 ± 3.99% to 43.15 ± 1.55%) and inhibition of tyrosinase (4.48 ± 1.50% to 40.53 ± 0.47%). The leaf extracts were not effective against butyrylcholinesterase, while 21.83 ± 3.82% and 36.80 ± 2.00% were the inhibitions demonstrated by the berry extracts (Romani et al. 2004).

Myrtle’s ability to protect the neuron in rats was studied against neurotoxicity induced with lipopolysaccharides (LPS), malondialdehyde, tumor necrosis factor α, nitric oxide, interleukin1β, Willebrand factor (VWF), asymmetric dimethyl arginine (ADMA), estrogen, 5LOX, 15LOX, and lipoxin A4. The tissue and serum of challenged rat’s brain were examined. The outcomes showed that investigated stress parameters significantly increased while estrogen level significantly decreased in rats intoxicated with LPS. Notable improvement was discovered in every studied biomarker (Shahmohammadi et al. 2012).

The central nervous effects of 80% *M. communis* leaf ethanolic extract (25–400 mg/kg, ip) were evaluated in mice and rats. Mice were subjected to grip strength, pentylenetetrazol-induced seizure, open field, and righting reflex assessments. Male rats were assessed for rapid eye movement (REM) as well as non-REM (NREM) sleep alterations. *M. communis* extract (50 – 200 mg/kg) assessment was done for horizontal activity (ED_50_ = 251 ± 55 mg/kg) and vertical activity (ED_50_ = 40.2 ± 6.6 mg/kg), while treatment with 200 and 400 mg/kg mitigated significant muscle tone in a method that is dependent on the dose. An important hypnotic, but no anticonvulsant effect, was recorded on administration with the extract at 200 mg/kg (*P*< 0.01). The reduced REM time of sleeping was shown by the electroencephalography results, while the NREM and total sleep times were significantly elevated compared to the mice control group (Aykac et al. 2019).

The mouse model was examined for the sedative-hypnotic effect of the aqueous extract. In the myrtle aqueous extract 200 mg/kg, the locomotor activity was significantly reduced in open field test (*P*<0.01). The hypnotic effect of aqueous extract of myrtle showed significance and was recorded in righting loss reflex test induced with pentobarbital induced (*P* <0.05) (EL-Kholy et al 2018). The efficacy of *M. communis* treatment on the factors that play a vital role in Alzheimer’s disease pathogenesis was compared with galantamine administration. The level of expression of muscarinic receptors M_1_, AchE, Ach activity, BDNF, GSH level, MPO, and MDA activity, and the gene expression of AchE were examined in rats induced with scopolamine. The results revealed that, *M. communis* administration in treatment significantly improved the reduction of latency in scopolamine-induced rats and object recognition time, elevating M_1_, BDNF, and Ach receptor levels of expression in the various regions of the brain. Furthermore, *M. communis* administration exhibited elevated AchE by improving GSH activity and decreasing MPO activity and MDA level (Naji et al. 2018).

## Protective effects

*M. communis* extract (300mg/kg bw, daily for 49 days) was studied for its protective effect against acrylamide and monosodium glutamate-induced hepatotoxicity in male rats. The results verified that the administration of monosodium glutamate and acrylamide lead to a significant elevation in LDL-C, TC, TL, TG, GGT, ALT, AST, ALP, TB, and MDA. However, myrtle leaves extract caused noticeable decrease in TP, GSH, Alb, TAC, CAT, SOD, GSH-Px, and HDL-C (Sen et al. 2016).

*M. communis* extract ameliorative effect was assessed on the arsenic chloride (AsCl_3_) toxicity-inducing tumor suppressor protein (P53) production in rats. The results showed that AsCl_3_ induced negative effects on the levels of P53-based gene expression and possibly P53 gene protein activity, bringing about low expression of gene. However, *M. communis* improved the status by elevating P53-based gene expression levels if used alone or mixed with AsCl_3_ (Samareh et al. 2018).

*M. communis* sp. was also examined for its antifibrotic and antioxidant effects against hepatic fibrosis and injury arising from biliary obstruction in rats. Direct bilirubin, plasma total bilirubin, alanine aminotransferase, aspartate aminotransferase, interleukin-1β levels, tumor necrosis factor-α were significantly elevated in the group with bile duct ligation, while these figures significantly declined in the bile duct ligation group administered with *M. communis* sp. *Communis* extract. Superoxide dismutase and glutathione were reduced significantly in the bile duct ligation group as compared to the groups in the control but increased significantly in extract-treated group. The levels of tissue luminol, myeloperoxidase activity, malondialdehyde, transforming growth factor-beta, lucigenin, and hydroxyproline increased dramatically in the bile duct ligation and were reduced in the group treated with the extract (Ozbeyli et al. 2020).

The therapeutic and protective effects of *M. communis* methanolic extracts (50 mg/kg/day, intraperitoneal ip, from days 0 to 13) and (50 mg/kg, ip, from 14 to 27 days), respectively, of the methanolic extract of *M. communis* were studied in rats against bleomycin-induced pulmonary fibrosis. Parenchymal inflammation and fibrotic changes, lipid peroxidation, and hydroxyproline content were significantly decreased in the myrtle extract-treated group, but catalase activity was greater. An enhancement in fibrosis and inflammation was seen in myrtle-treated group, particularly in the initial fibrosis phase (preventive regime) (Mahboubi et al. 2016).

*M. communis* ssp. leaf ethanol extract protective effect administration (for 14 days before induction) was studied against acute pancreatitis induced with cerulenin in rats. Pancreatic damage induced by cerulenin was related with elevated serum activity of amylase and lipase, the pancreatic activity, level of myeloperoxidase, malondialdehyde, interleukin-1β, and interleukin-6. Acute pancreatitis similarly manifested by a decline in the levels of anti-inflammatory interleukin-10 and glutathione in the pancreas. In the extract pretreatment group, before the induction of pancreatitis, the pancreatic injury detected through the histological analysis was significantly reduced, including a reversal in biochemical changes (Zohalinezhad et al. 2016).

## Gastrointestinal effects

*M. communis* essential oil and its decoction reduced the average time of pain and lessened ulcers size in patients with slight recurring aphthous stomatitis, showing no antagonistic effects. All the patients were content with *M. communis* topical essential oil (5%); 81% of patients preferred *M. communis* topical decoctions (5%). The effectiveness of *M. communis* was linked to its antiseptic, anti-inflammatory, analgesic, and its wound healing effects (Jabri et al. 2016a, b, c).

The aqueous seed extract of myrtle berry’s protective effects against induced esophageal reflux was studied in rats. The induced esophageal reflux caused noticeable histopathological and macroscopic variations in the esophagus. It also followed oxidative stress as measured by a rise in lipid peroxidation, a reduction of the sulfhydryl groups, and levels of glutathione, including the depletion of antioxidant enzyme activities. The extract nullified all biochemical, histopathological, and morphological changes. The induced esophageal reflux also amplified esophageal calcium, free iron levels, and hydrogen peroxide, while extract management protected compared to intracellular mediator’s deregulation (Bouzabata 2013).

*M. communis* effects in gastroesophageal reflux disease were investigated in comparison with omeprazole through 42 days double-blind randomized controlled scientific trial. About 45 applicants were randomly given three groups: myrtle berries freeze-dried aqueous extract (1000 mg/day), omeprazole capsules (20 mg/day), and the third group received a combination of both treatments. The dyspeptic and reflux scores decreased significantly in all groups when compared with the respective standards. However, substantial changes existed in myrtle extract group (acid reflux and dysmotility-like symptoms related scores) (Benchikh et al. 2016).

The anti-gastric ulcer effect of *M. communis* dried berry aqueous extract (105 and 175 mg/kg, orally) and methanolic extract (93 and 154 mg/kg, orally) was studied against rats induced with pyloric ligation, ethanol, and indomethacin. Aqueous extract administered orally (at both doses) caused the ulcer index in all prototypes of ulcers to decrease significantly. The aqueous extract (small dose) and methanolic extract (high dose) of *M. communis* demonstrated effects that were more significant than those of omeprazole in ulcer models induced with ethanol. Two doses of extracts lessened gastric juice volume and total acidity but elevated gastric wall mucus content and gastric pH in the ulcer’s models. Histopathological studies of rat’s gastric tissues administered with methanolic and aqueous extracts in ulcer induced with indomethacin exhibited a significant protective effect against ulcer at that dose level (Sumbul et al. 2012).

The effects of *M. communis* berry juice (5 and 10 ml/kg bw, orally) on gastric emptying and normal gastro-intestinal transit, including diarrhea induced by castor oil, oxidative stress in the small intestine, and entero-pooling tests, were studied in rats. The juice inhibited intestinal motility and gastric emptying in a dose-dependent manner. The juice administration equally caused a significant dose-dependent defense against diarrhea and intestinal fluid buildup. The status of oxidative stress in the intestine associated with intestinal hypersecretion induced with castor oil was attenuated via juice administration (Jabri et al. 2016a, b, c).

## Antidiarrheal effects

In mice, model-treated *M. communis* essential oils were examined for their antidiarrheal and antisecretory activities. The *M. communis* oil [abundant in 1.8-cineole 26.5% and α-pinene 54.1%] reduced significantly the gastric emptying dose of 500 mg/kg and the intestinal transfer at the entire doses utilized (50, 250, and 500 mg/kg). The extract essential oil similarly possessed anti-diarrheal and anti-secretory action depending on the dose (Sisay et al. 2017).

The antidiarrheal effects of an aqueous extract of *M. communis* berry seeds were studied in diarrhea induced with castor oil in rats. The defense of the extract depended on the dose against intestinal fluid and diarrhea buildup. Hypersecretion of the intestine induced with castor oil was complemented by the intestinal status of oxidative stress; hydrogen peroxide in the intestine also increased, as did free iron and calcium levels; however, the group pre-treated with the extract showed alteration in all castor oil-induced intracellular mediators’ turbulence. The results also showed that the extract has abundant total and condensed tannins and presents antibacterial action in a broad spectrum (Panahi et al. 2014).

The methanol extracts of 200 mg/kg (*P*< 0.05) and 400 mg/kg (*P*< 0.01), including the methanol and chloroform fractions of 400 mg/kg (*P*< 0.05), all significantly hindered the commencement of diarrhea. All doses of methanol extract, and both segments at 300 and 400 mg/kg, expressively lessened the regularity and mass of fecal yields. Charcoal meal test output showed the methanol extract at all prescriptions (*P*< 0.001) as well as the two fractions of 300 mg/kg (*P*< 0.05) and 400 mg/kg produced a significant (*P*< 0.001) anti-motility effect. In the entero-pooling test, the methanolic extract at all verified prescriptions (*P*< 0.01), and the fractions at 300 mg/kg and 400 mg/kg (*P*< 0.05) produced a notable decrease in the capacity and mass of intestinal substances (Mahboubi et al. 2017).

## Anti-hemorrhoid effects

The efficiency of topical cream from the essential oil *M. communis* in alleviating hemorrhoids symptoms compared to ointments that are anti-hemorrhoid (containing zinc oxide, lidocaine, hydrocortisone, and aluminum subacetate) was evaluated in a random double-blind double-dummy trial performed on 106 patients. All assessed indications (long-lasting pain, bleeding, pain during excretion, and irritation, anus itching, tenesmus, and heaviness) were considerably diminished by trial end (*P*<0.001). No remarkable difference was noticed in the level of improvement of evaluated symptoms concerning ointment of anti-hemorrhoid and *M. communis* (*P*>0.05) (Malekuti et al. 2019).

The therapeutic effect of the essential oil of *M. communis* on hemorrhoids management was clinically examined. The results pointed out that the essential oil of *M. communis* (cream or ointment) significantly ameliorated hemorrhage, excretion pain, perpetual pain, anal itching, anal irritation, and anal weightiness in types I and II hemorrhoids’ patients. *M. communis* was dynamic in patients’ treatment, not responding to organic product usages (anti-hemorrhoids cream) (Janbaz et al. 2013).

A triple-blind random controlled trial was done to regulate the anti-hemorrhoid effect of *M. communis* ointments (two times a day, every single 12 ± 2 h, through the rectum for 28 days) compared with anti-hemorrhoid ointment, taking the life quality and hemorrhoid into consideration (main outcomes) with contentment of the management and harmful effects (minor results) in females with grade I and II hemorrhoid. The colorectal assessment of a clinical therapeutics gauge (CORECTS) was utilized to measure the severity of hemorrhoid and the World Health Organization Life Quality Survey (WHOQOL-BREF) to decide the value of life. The harshness of hemorrhoid symptoms was reduced in both groups, showing no significant difference statistically between both groups (*P*>0.05). The anal itching mean was significantly lower 28–56 days after involvement in the *M. communis* cream group (*P*<0.05). No significant difference was seen between groups at 28–56 days after considering life quality (*P*>0.05). The participants given *M. communis* cream remained significant, showing satisfaction in the drug usage (Harassi et al. 2019).

## Effects on smooth muscles

M. communis crude methanol extract triggered full contractions induced by K^+^ (80 mM) that are also spontaneous in isolated rabbit jejunum. It induced right-side shift similar to response curves of calcium. M. communis crude methanol extract also caused relaxation on K^+^ (80 mM)-induced contractions in isolated rabbit tracheal arrangements and caused phenylephrine relaxation (1 μM) and K^+^ (80 mM)-induced contractions in isolated rabbit aorta preparations, like verapamil, a standard calcium channel blocker (Ozturk and Duran 2018).

## Anticancer and antimutagenic effects

The cytotoxicity of *M. communis* oils was investigated against MCF-7 and P815 cells. P815 cells showed less activity than MCF-7 cells, with IC_50_ values of 4–6.25 µg/ml against the essential oils. Cell toxicity is related to DNA fragmentation, an important apoptosis hallmark (Tretiakova et al. 2008).

The cytotoxic effect of water, ethanol, methanol, n-hexane, and ethyl acetate extracts of *M. communis* leaves was assessed in vitro against brine shrimp. All the extracts showed cell toxicity against brine shrimp (LC_50_ < 1000). The extracts of *n*-hexane extracts and ethyl acetate showed additional cytotoxic activity compared to other extracts (Fani et al. 2014). The essential oil of *M. communis* antiproliferative effect was studied against cancer Vero cells and HCT 116. *M. communis* IC_50_ values were examined at 25 μg/ml in HCT 116 and 15 μg/ml in Vero cells (Hayder et al. 2008).

The anticancer potential of myrtucommuacetalone-1 (MCA-1), isolated from *M. communis*, was assessed with an MTT cytotoxic assay. Cytotoxicity investigations of MCA-1 on 3T3 mouse fibroblasts, CC1 liver cell line, J774.2, macrophages, and MDBK bovine kidney epithelial cell revealed IC_50_ values of 6.53 ± 1.2, 4.6 ± 0.7, 5 ± 0.8, and 4.6 ± 0.7, µg/ml, respectively (Maxia et al. 2011).

Myrtucommulone strongly induced the death of cells of diverse cancer cell lines (EC_50_ 3-8 microM); apoptosis was visualized by stimulating cleavage of poly (ADP-ribose) polymerase (PARP) cleavage, caspase-3, -8, and -9, DNA fragmentation, and nucleosomes release into the cytosol. It was not cytotoxic against foreskin fibroblasts with EC_50_ of cell death around 20–50 microM or non-transformed human peripheral blood mononuclear cells (PBMC). Furthermore, its apoptotic effects were facilitated by the intrinsic, not extrinsic death pathway. It triggered the reduction of mitochondrial membrane potential in MM6 cells, and mitochondria cytochrome c release is induced (Choudhary et al. 2013).

*M. communis* aqueous, chloroform, ethyl acetate, methanol, hexane, total flavonoids oligomer fraction, and essential oil significantly reduced the response to DNA damage caused by nifuroxazide and aflatoxin B1 in a system of bacterial assays (*Escherichia coli* PQ 37 with the SOS chromotest). The extracts of methanol and ethyl acetate displayed strong inhibition against DNA damage response caused by secondary genotoxic aflatoxin B1 (Ghadami et al. 2014).

*M. communis* oils antimutagenic properties were assessed against reactive mutagenesis induced by t-BOOH in *Escherichia coli* oxyR mutant IC 202, (a bacterial strain) unable to eliminate ROS. A decline in reactive mutagenesis occurred when myrtle oil was administered with the highest concentration at 13%. During the use of oxidative mutagen, the oil greatly reduced mutagenesis in a manner dependent on the concentration (at the highest concentration of 28%) (Cottiglia et al. 2012).

## Immunomodulatory effect

Myrtucommuacetalone, myricetin, myrtucommulone M, myrtucommulone E isousnic acid, and growth regulator G3 factor are all* M. communis* isolates assessed for their ability to lessen the immune response through their effects on various immune system constituents. Compounds of myrtucommuacetalone and growth regulator G3 factor were significantly inhibited against nitric oxide (NO) production. Myrtucommuacetalone also revealed important antiproliferation (IC_50_< 0.5 μg/ml) against proliferation of T-cell. Significant inhibition was caused by myricetin (IC_50_ = 1.6 μg/ml) on the blood phagocytes stimulated by zymosan in ROS generation. The compounds myrtucommuacetalone and myricetin were active against ROS generation stimulated by PMA (Ozcan et al. 2019).

## Dermatological effects

*M. communis* can be applied topically in wart treatment. It displayed an additional rapid response compared to salicylic acid and very few side effects (Salehpour et al. 2016).

The protective effect of oral or topical *M. communis* management (100 mg/kg per day) either topically or orally for 2 days was investigated against rats induced with burn damage (in the burn group, dorsum was clean-shaven and kept in water bath with a temperature of about 90 °C). The burn injury in the skin was triggered by heat, causing a significant reduction in glutathione and nitric oxide levels and activities of catalase, superoxide dismutase, and tissue factor, followed by a significant increase in skin malondialdehyde levels. Myrtle management altered every biochemical index and also caused histopathological alterations as a result of heat trauma. The topical and oral administration of myrtle extract possessed an ameliorative role in the oxidative damage induced by the wound (Hayder et al. 2008).

## Toxicity and side effects

The ethanolic and aqueous extracts of aerial parts of *M. communis* showed LD_50_ values of 0.79 and 0.473 g/kg in mice separately (Soomro et al. 2019).

In Arabi sheep, blood parameters were assessed for the effect of *M. communis* leaf on them. The result showed significant reduction in the glucose, triglyceride, and blood urea of sheep when fed a diet containing 4% myrtle leaf compared with the control diet (D’Urso et al. 2017).

Recent studies that elaborated on various aspects of the therapeutic potential of *M. communis* include Dabbaghi et al. (2023) that states that myrtle’s strong antioxidant concentration is one of its most important defensive qualities. Research have demonstrated that myrtle’s antioxidant qualities can offer defense against dangerous compounds like pesticides, heavy metals, and other pollutants found in the environment. Furthermore, myrtle possesses anti-inflammatory qualities that may lessen the harm brought on by prolonged exposure to pollutants. Myrtle’s anti-inflammatory and antibacterial qualities have also shown promise in treating gastrointestinal disorders like stomach ulcers.

In fact, product from *M. communis* is getting closer to the clinic as a randomized clinical trial was carried out to assess the therapeutic effects of *M. communis* on chromic skin lesions, and they measured the duration of sleep, number of nighttime awakenings, quality of life, and chronic skin problems and itching-related characteristics (such as the itching time, severity, distribution, frequency, and calculated itching score) in the two groups. Applying myrtle cream effectively reduced skin issues, such as itching and burning feeling, according to our data research. Furthermore, when compared to pre-treatment, myrtle significantly reduced skin lesion symptoms such as excoriation in the case group. Myrtle cream was found to have a notable positive impact on the patients’ quality of life within the case group. More detailed information about the preventive effect of myrtle against skin complications caused by sulfur mustard is provided by this study. Additionally, myrtle significantly raised the veterans’ quality of life who had been exposed to sulfur mustard (Iman et al. 2022).

## Conclusion

*M. communis* is used at different places throughout the world as a traditional herbal remedy. It contained many bioactive ingredients and possessed a wide range of pharmacological effects like anti-inflammatory, analgesic, antimicrobial, antiparasitic, antioxidant, antidiabetic, cardiovascular, central nervous, protective, gastrointestinal, anticancer, antimutagenic, immunomodulatory, dermatological, and many other biological effects. According to the data on in vitro and in vivo studies, *M. communis* has the potential to be used in pharmaceutical development as a medicine for wound healing, treatment of gastrointestinal disorders, and cancer management, to name a few, because of its safety and effectiveness.

## Data Availability

Data sharing not applicable to this article as no datasets were generated or analyzed during the current study.
